# Progress in mechanochemical synthesis of catalysts for the CO_2_ processes: a step towards carbon neutrality

**DOI:** 10.1039/d5ra09717g

**Published:** 2026-02-04

**Authors:** Anam Shahzadi, Muhammad Adnan Iqbal, Adnan Majeed, Iqra Yasmeen, Manahil Akmal, Sana Ejaz, Sabahat Fatima, Muhammad Nadeem Arshad, Mohammad Asad

**Affiliations:** a Department of Chemistry, University of Agriculture Faisalabad Faisalabad-38000 Pakistan adnan.iqbal@uaf.edu.pk; b Organometallic and Coordination Chemistry Laboratory, University of Agriculture Faisalabad Faisalabad-38000 Pakistan; c Department of Chemical Engineering, Laval University Quebec QC G1V 0A6 Canada; d Center of Excellence for Advanced Materials Research (CEAMR), King Abdulaziz University Jeddah 21589 Saudi Arabia

## Abstract

This review examines mechanochemical synthesis as a solvent-free and scalable approach for high-performance CO_2_ reduction catalysts. Mechanical energy-driven methods, particularly high-energy ball milling, enable controlled defect formation, enhanced metal-support interactions, and atomic dispersion, thereby improving CO_2_ activation and conversion. Recent advances are discussed across major reaction pathways, including methanation (>99% CH_4_ selectivity), light olefin production (55.4% selectivity), photocatalytic CO_2_ reduction (CO production up to 306.1 µmol g^−1^ h^−1^), and integrated capture utilization systems. Structure performance relationships and techno-economic aspects are evaluated, highlighting 60–90% waste reduction and 20–50% energy savings relative to conventional synthesis. Overall, mechanochemistry represents a versatile and scalable platform for advancing sustainable CO_2_ valorization strategies.

## Abstract 2

This review highlights mechanochemical strategies for CO_2_ reduction catalysis, enabling sustainable pathways toward carbon-neutral products and environmental remediation.

## Graphical abstract

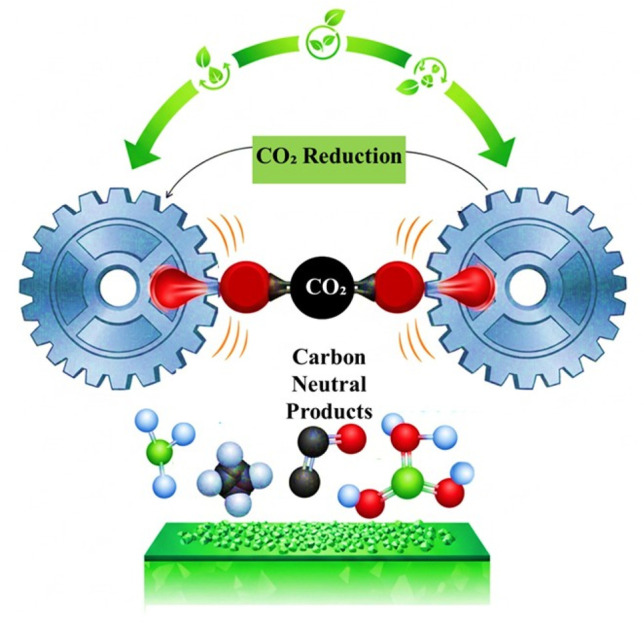

## 1 Introduction

The continuous rise in atmospheric carbon dioxide (CO_2_) levels due to anthropogenic activities has posed a formidable challenge to global climate systems.^1^ As a major greenhouse gas, CO_2_ contributes significantly to global warming, prompting urgent research into effective mitigation strategies.^2^ Over the last 170 years, human-induced CO_2_ emissions have sharply increased atmospheric CO_2_ concentrations from pre-industrial levels of ∼280 parts per million (ppm) to approximately 417 ppm in 2024, as recorded by the Mauna Loa Observatory.^3^ If current CO_2_ emission trends persist, atmospheric CO_2_ could exceed 450 ppm by 2030, greatly increasing the risk of severe climate impacts such as intensified heatwaves, rising sea levels, and widespread disruptions to ecosystems and food security.^4^ These alarming trends have intensified the demand for efficient carbon management strategies that can both mitigate emissions and transform CO_2_ into useful products. Among the various approaches, the catalytic hydrogenation of CO_2_ into valuable chemicals such as methane and light olefins has emerged as a favorable solution.^5^ This transformation not only helps reduce greenhouse gas emissions but also enables the sustainable production of chemical feedstocks, thereby supporting a circular carbon economy.^6^

One of the critical factors influencing the efficiency of CO_2_ reduction is the nature of the catalyst and its preparation method.^7^ Traditional synthesis techniques often rely on high temperatures and solvents, which may lead to environmental and scalability challenges.^8^ In contrast, mechanochemistry, a solvent-free, energy-efficient, and scalable approach, has attracted considerable attention for catalyst fabrication. Mechanochemistry, originally introduced by Wilhelm Ostwald in 1919, involves inducing chemical transformations through mechanical forces such as grinding or high-energy ball milling.^9^ These processes cause physical changes like fracturing, welding, and re-welding of materials, resulting in enhanced surface area, reduced crystallite size, and increased surface defects, all of which contribute to improved catalytic activity.^10^

In the context of catalytic material development, mechanochemical synthesis is capable of producing nanostructured catalysts with distinctive features such as high defect densities, amorphous or nanocrystalline phases, abundant oxygen vacancies, and tailored surface chemistry.^11^ These structural modifications significantly enhance CO_2_ adsorption and activation, critical steps in the CO_2_ reduction mechanism.^12^ Moreover, the method facilitates the incorporation of heteroatoms, formation of heterostructures, and creation of strong metal-support interactions, all of which improve electron transfer, promote hydrogen dissociation, and ensure homogeneous dispersion of active metal species.^13^ Beyond these benefits, mechanochemistry offers compatibility with a wide range of materials, from metals and metal oxides to carbides, nitrides, and hybrid composites, enabling precise tuning of catalytic functionality.^14^ Within the broader landscape of CO_2_ conversion, mechanochemistry complements other established strategies such as electrochemical, thermal, photothermal, photocatalytic, and biological routes that aim to transform this stable molecule into value-added products.^15^

**Fig. 1:**
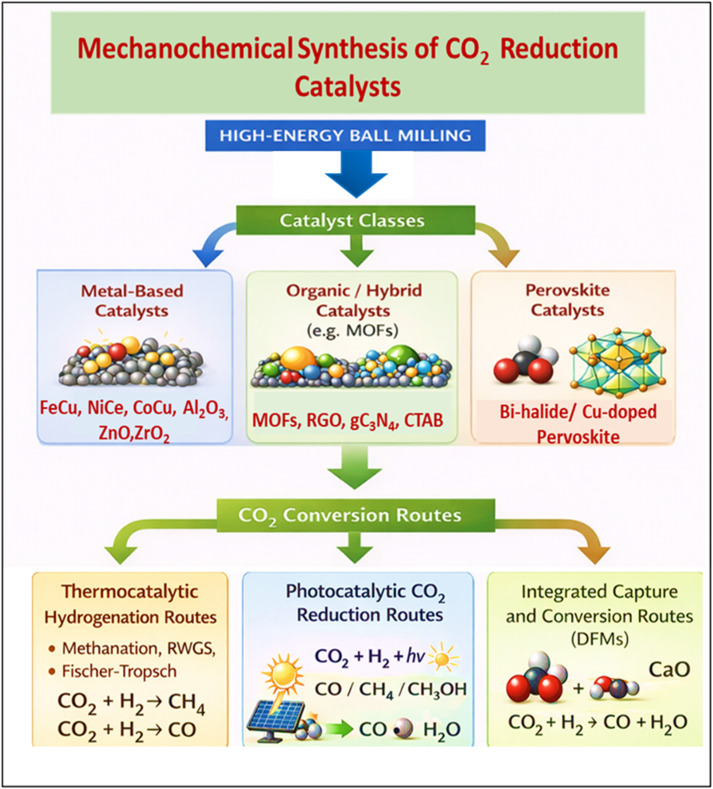
Schematic representation of mechanochemical synthesis routes and CO_2_ conversion pathways.

This versatility makes mechanochemistry particularly well-suited for CO_2_ reduction applications.^16^ Mechanochemically synthesized catalysts have demonstrated notable improvements in selectivity, activity, and stability under both low and high temperature operating conditions (Fig. 1). Their unique microstructural and electronic features open new avenues for tuning active sites toward desired products, whether in methanation or light olefin synthesis. This review provides an in-depth examination of recent advances in mechanochemically synthesized catalysts for CO_2_ reduction, with particular attention to methanation and olefin production. It synthesizes insights from synthesis strategies, structural and physicochemical characterizations, and mechanistic studies, linking these to catalytic performance trends. By highlighting the correlations between mechanochemical processing parameters and the resulting catalyst functionalities, this work aims to establish clear structure–activity relationships. In doing so, it not only underscores the scientific progress in mechanocatalysis but also identifies knowledge gaps and future research directions needed to translate laboratory successes into industrial-scale CO_2_ utilization technologies.

## 2 Mechanochemically synthesized catalysts for CO_2_ reduction

### 2.1 Catalysts for CO_2_ hydrogenation to fuels and chemicals

Depending on catalyst composition and reaction conditions, CO_2_ hydrogenation can yield a range of C1 products, including CO, CH_4_, and CH_3_OH, as well as C2 and higher hydrocarbons and oxygenates, such as C_2_H_4_, C_2_H_6_, and ethanol (C_2_H_5_OH), which are highly attractive as fuels and chemical feedstocks. Among these, light olefins (C_2_–C_4_) are essential building blocks for the polymer and petrochemical industries, while methane remains a valuable energy carrier. However, CO_2_ is a thermodynamically stable and chemically inert molecule (Δ*G*°_(298.15 K)_ = −394.4 kJ mol^−1^), making its activation particularly challenging.^17,18^ Early catalytic strategies for CO_2_ hydrogenation focused on two principal pathways: the direct methanol synthesis route and the tandem reverse water gas shift (RWGS) reaction followed by Fischer Tropsch synthesis (FTS).^19^ In the latter pathway, CO_2_ is first converted to CO *via* the endothermic RWGS reaction (Δ*H*°_(298.15 K)_ = +41 kJ mol^−1^), followed by exothermic C–C coupling through FTS (Δ*H*°_(298.15 K)_ = −152 kJ mol^−1^) to form C_1_–C_2_^+^ hydrocarbons and oxygenates, attracting significant research attention.^20^

#### 2.1.1 Iron-based catalysts for light olefin synthesis

Iron-based catalysts are widely used for CO_2_ reduction due to their dual functionality in both reactions. However, product selectivity is often limited by the Anderson–Schulz–Flory distribution, restricting light olefin selectivity to about 58%.^21^ To overcome these limitations and create more efficient catalysts, researchers have increasingly turned to mechanochemical techniques, particularly high-energy ball milling. This method is used to engineer catalysts with enhanced properties like improved surface area, smaller crystallite size, and higher defect density. One successful application involves modifying iron-based catalysts for light olefin synthesis. By using ball milling, researchers can promote the formation of active heterostructures, such as O–Fe/Mg–O, when magnesium is used as a promoter.^22^ The addition of potassium further enhances chain growth during Fischer–Tropsch synthesis while suppressing unwanted methane formation.^23^ These modifications have enabled Fe–Mg catalysts to achieve CO_2_ conversions of 32.1% and light olefin selectivity of 55.4%, demonstrating the power of mechanochemical engineering in sustainable CO_2_ valorization.^24^ However, the heterogeneity of mechanical forces during ball milling can lead to batch-to-batch variations in particle size distribution and active phase dispersion, complicating industrial scale-up. Additionally, iron-based catalysts are prone to carbide formation and sintering under harsh reaction conditions, which can gradually reduce selectivity toward light olefins. The long-term stability under realistic flue gas conditions (containing SO_2_, H_2_O, and particulates) requires further investigation, as trace impurities may poison active sites or alter the Fe–Mg interfacial structure.^25^

#### 2.1.2 Nickel-based catalysts for CO_2_ methanation

Mechanochemical methods have also proven highly effective in the catalytic hydrogenation of CO_2_ to methane (CH_4_), a reaction first reported by French chemist Paul Sabatier. The Sabatier reaction is known to proceed through multiple mechanistic pathways involving intermediates like CO*, HCOO*, or COOH*.^26^ Nickel-based catalysts, often supported on oxides like Fe_3_O_4_ or MgO, are well-known for their excellent activity and thermal stability for this reaction.^27^ Recent advances have further refined catalyst design using mechanochemistry. The development of a Ni-incorporated MgO/MgH_2_ catalyst is a prime example.^28^ Synthesized through high-energy ball milling under a hydrogen atmosphere, this catalyst achieved remarkable CO_2_ conversion (85.2%) and CH_4_ selectivity (99.5%) at 300 °C and 1.0 MPa.^29^ This success was attributed to the formation of a unique “Mg–Ni–O” heterostructure, which significantly improves CO_2_ chemisorption and H_2_ dissociation.^30^ Mechanistic studies show that the surface hydrogen species generated play a crucial role: electropositive H^+^ species favor the O-terminal hydrogenation pathway (COOH*), while electronegative H^−^ species promote the C-terminal hydrogenation pathway (HCOO*). This highlights how mechanochemical methods can strategically influence reaction pathways to boost efficiency.^31^

#### 2.1.3 Bimetallic catalysts for enhanced performance

Another favorable avenue is the use of bimetallic catalysts, which are known for their enhanced catalytic activity and stability compared to monometallic systems.^32^ Ni–Co bimetallic catalysts, in particular, have shown great potential for CO_2_ methanation, achieving a CO_2_ conversion of 84.5% and CH_4_ selectivity of 99.8% at low temperatures. Structural analysis revealed that ball milling leads to a more uniform distribution of Ni species around the Co species. While the Co/SiO_2_ catalyst shows stronger CO adsorption and the Ni/SiO_2_ catalyst is superior in H_2_ dissociation, the Ni_2_Co_3_/SiO_2_ catalyst synthesized mechanochemically exhibits excellent performance in both H_2_ dissociation and CO adsorption.^33^ This was attributed to the transfer of electrons from Ni to Co, which forms electron-rich Co species that enhance CO_2_ adsorption and promote H_2_ dissociation through both electron donation (Co 4s, 3p) and back-donation (Co 3d). Despite these auspicious results, a deeper understanding of their catalytic mechanisms is needed to fully optimize their application.^34^

While bimetallic systems show enhanced activity, the precise control of metal ratios and intermetallic phase formation during ball milling remains challenging. The synergistic effects are highly sensitive to synthesis conditions (milling time, speed, atmosphere), and deviations can result in phase segregation or formation of inactive alloys.^35^ Moreover, under reducing atmospheres at elevated temperatures, the bimetallic structure may undergo reconstruction, potentially losing the beneficial electronic interactions. *Operando* spectroscopic studies are needed to monitor the evolution of active sites during reaction and understand deactivation mechanisms.^36^

#### 2.1.4 Effect of support materials and promoters

Beyond bimetallic systems, the performance of Ni-based catalysts, widely recognized for their affordability and abundance, is significantly influenced by the support material.^37^ Supports such as Al_2_O_3_, ZrO_2_, SiO_2_, and Cr_2_O_3_ impact the dispersion of active species and metal-support interactions, which are crucial for CO_2_ adsorption and overall catalytic performance.^38^ Chromium oxide (Cr_2_O_3_), for example, is an effective material that has shown success in various reactions, including toluene oxidation and Fischer–Tropsch synthesis.^39^ The synthesis method is also a critical factor in optimizing catalytic properties. While traditional techniques like co-precipitation and impregnation are used, mechanochemical synthesis offers a solvent-free, scalable, and eco-friendly route.^40^ Studies have shown that adding promoters like Mn, La, Ca, Co, and Cu can enhance stability, dispersion, and reducibility. For instance, a Ni/Cr_2_O_3_ catalyst promoted with Mn and La, prepared mechanochemically, demonstrated enhanced CO_2_ methanation performance. The catalyst with 15 wt% Mn showed the highest CO_2_ conversion (72.12%) and 100% CH_4_ selectivity at 350 °C, and maintained stability over 12 hours. This highlights how promoters and precise control of synthesis conditions, such as calcination temperature, are vital. A higher calcination temperature, for example, can decrease the surface area and reducibility while increasing NiO crystallite size, which ultimately reduces catalytic activity.^41^

A new study on Ni/Al_2_O_3_ catalysts for CO_2_ methanation further highlights the advantages of mechanochemistry. This solvent-free, scalable, and energy-efficient route was used to prepare catalysts with varying Ni loadings (5–20 wt%) *via* simple dry milling.^42^ Structural and catalytic analysis identified 15 wt% Ni as the optimal composition, achieving 75.2% CO_2_ conversion and 96.8% CH_4_ selectivity at 400 °C. The 15Ni/Al_2_O_3_ sample exhibited a favorable mesoporous structure and a high surface area, which are crucial for promoting CO_2_ adsorption and hydrogenation. Importantly, calcination at 400 °C was found to be critical for preserving small Ni particles and high surface area, while higher temperatures promoted the formation of inactive Ni/Al_2_O_3_ spinel. The robustness of catalyst was confirmed across varied operating conditions, demonstrating its potential for carbon-neutral energy systems.^43^

While these Ni-based catalysts show excellent laboratory performance, several practical issues must be addressed for industrial implementation. The mechanical milling process requires careful optimization of ball-to-powder ratio, milling atmosphere (inert *vs.* reactive), and post-treatment conditions.^11^ The energy input for prolonged milling at industrial scale may offset some of the environmental benefits compared to conventional wet chemistry routes.^11^ Furthermore, nickel is susceptible to poisoning by sulfur compounds commonly present in industrial CO_2_ streams, necessitating either stringent feed purification or development of sulfur-tolerant formulations. The thermal management of highly exothermic methanation reactions in large-scale reactors also presents engineering challenges.^44^

#### 2.1.5 Manganese oxide catalysts

A novel, one-pot mechanochemical synthesis route has also been developed for fabricating manganese oxide (MnO_*x*_) nanostructures, including Pt and Cu-doped variants, for catalytic CO_2_ hydrogenation. The milling speed was found to be a crucial factor in tuning the crystal structure, porosity, and surface area, which directly correlated with catalytic performance. The MnO_*x*_ catalyst milled at 600 rpm showed the highest activity at 823 K, attributed to a favorable mix of Mn(ii), Mn(iii), and Mn(iv) oxidation states.^45^ Doping with Pt and Cu further boosted activity, with Pt-doped systems achieving a 12–13-fold increase. Interestingly, at higher temperatures, the distribution of manganese oxidation states became more critical than the specific dopant, showing the dominance of the mechanochemically modified surface.^46^

#### 2.1.6 Multi-metallic catalysts for alcohol synthesis

The development of multi-metallic K-doped Cu–Fe/ZnO–Al_2_O_3_ (KCFZA) catalysts *via* distinct ball milling strategies also highlights mechanochemistry's powerful influence on catalytic performance for CO_2_ hydrogenation to alcohols.^47^ While a conventional solution-based method (SS1-K) yielded nanocrystalline platelets, the ball milling methods produced smaller, more spherical particles and were significantly more sustainable, with one method (ball milling) achieving an E-factor of 0.33 and 86% energy savings.^48^ These mechanochemical routes not only improved atom economy but also allowed for a tuning of product selectivity, with different milling strategies favoring methanol, ethanol, or acetic acid.^49^

#### 2.1.7 Copper–zirconia systems for methanol synthesis

Mechanochemistry's versatility extends to the synthesis of a variety of new catalytic systems. For methanol synthesis, a cost-effective and scalable approach involves the direct physical mixing of sponge copper (SC) and amorphous zirconia (a-ZrO_2_).^50^ This method, particularly with planetary ball milling, creates robust Cu–ZrO_2_ interfacial sites that are highly active for CO_2_ hydrogenation. The amorphous nature of the ZrO_2_ minimizes methanol decomposition, and the process promotes a beneficial structural collapse of the copper, leading to high methanol production rates under mild conditions. This contrasts with traditional calcination-based methods, which often have higher energy demands and stability issues.^51^

#### 2.1.8 MOF-derived catalysts

The post-synthetic modulation of metal–organic frameworks (MOFs) represents a novel strategy for designing catalysts that goes beyond traditional reliance on high surface area and porosity. MOFs, with their tunable metal nodes and organic linkers, offer a versatile platform for CO_2_ adsorption and activation, as well as for generating highly dispersed active sites upon conversion to derivatives. A notable example is bimetallic ZnCu-MOF-74, which initially exhibits moderate catalytic activity. When subjected to mechanochemical amorphization, the framework's crystallinity collapses and porosity decreases; however, the catalytic performance is enhanced. This counterintuitive improvement arises from the creation of additional accessible active sites *via* controlled cleavage of metal–carboxylate bonds, effectively decoupling porosity from catalytic function and yielding selectivity comparable to industrial Cu/ZnO/Al_2_O_3_ catalysts.^52^ Recent studies highlight that post-synthetic modifications, including heteroatom incorporation, defect engineering, or partial amorphization, can significantly improve active site dispersion, electronic properties, and stability, facilitating efficient CO_2_ activation and selective hydrogenation to fuels and chemicals {Ali, 2025 #485}{Qureshi, 2025 #486}. These findings underscore a new paradigm in which MOF-derived catalysts, prepared *via* mechanochemistry or other post-synthetic approaches, provide tunable structural and electronic environments that enhance catalytic performance even when traditional structural features, such as crystallinity or high porosity, are intentionally disrupted.

#### 2.1.9 Sodium-promoted Fe–Cu catalysts for C_2_^+^ hydrocarbons

**Fig. 2:**
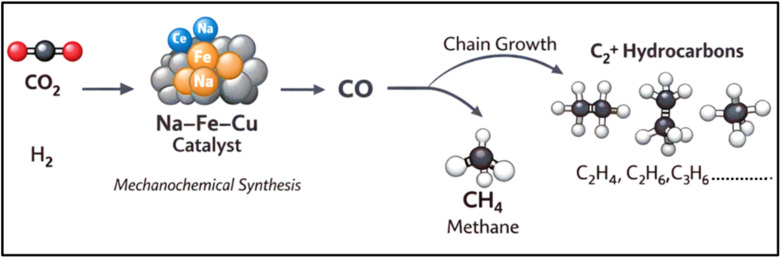
CO_2_ hydrogenation to C_2_^+^ hydrocarbons over a Na–Fe–Cu mechanochemical catalyst.

For the synthesis of multi-carbon (C_2_^+^) hydrocarbons, a Na-promoted Fe–Cu catalyst was fabricated *via* a sustainable, solvent-free mechanochemical route. This catalyst achieved a high C_2_^+^ selectivity of 88.8% by leveraging synergistic effects from Na and Cu, which improved iron reducibility and facilitated H_2_ activation (Fig. 2). The method's complete elimination of liquid waste and improved control over microstructure offer significant advantages over wet chemistry, establishing a green, scalable route for sustainable synthetic fuel technologies.^53^

#### 2.1.10 Single-atom catalysts on metal oxides

Single-atom catalysts (SACs) have emerged as a transformative catalyst platform in which isolated metal centers anchored on solid supports enable maximized atom utilization, tunable electronic structures, and distinct reaction pathways compared to nanoparticulate systems.^54^ Mechanochemical synthesis has gained increasing attention as a sustainable and scalable route for SAC fabrication, offering precise control over atomic dispersion and metal-support interactions without the need for solvents or high-temperature treatments. Using this approach, Pd and Cu single atoms uniformly dispersed on TiO_2_ have demonstrated markedly enhanced CO_2_ photoreduction activity up to 11-fold higher than pristine TiO_2_ (271.6 µmol g^−1^ h^−1^) which has been attributed to strong Pd–CO_2_ binding that effectively lowers the activation energy barrier for CO_2_ conversion.^55^

Beyond photoreduction, Cu-based SACs supported on ZnO have shown exceptional performance in electrochemical CO_2_ reduction, where positively charged isolated Cu sites promote efficient CO_2_ adsorption and activation while stabilizing key *OCHO intermediates during the rate-limiting first protonation step, thereby facilitating multistep proton-coupled electron transfer toward value-added products such as formate and alcohols. Recent studies further reveal that SAC performance can be amplified through cooperative catalytic effects in complex oxide systems, including perovskite-derived catalysts undergoing electrochemical reconstruction, where dynamically generated active sites and mobile A-site cations enhance CO_2_ activation while suppressing competing hydrogen evolution reactions.^56^ Collectively, these advances underscore the critical importance of atomic-level structural control, synergistic metal-support interactions, and dynamic catalyst evolution. Mechanochemical synthesis, combined with *in situ* reconstruction strategies, thus represents a powerful and environmentally benign toolbox for designing next-generation SACs for efficient and selective CO_2_ conversion, with significant implications for sustainable chemical production and environmental remediation.^57^

#### 2.1.11 SACs for CO oxidation

Beyond CO_2_ reduction, mechanochemically prepared SACs are also proving to be highly effective for CO oxidation, addressing a major environmental and health concern. Noble metal SACs (Au, Pt, Pd, Rh) anchored on ZnO have shown outstanding activity at room temperature.^58^ While some SACs, like Au_1_/ZnO, can suffer from atom aggregation and deactivation over time, other systems have been optimized for stability and performance. For example, Rh_1_/ZnO nanowires demonstrated superior catalytic activity for CO oxidation due to a low energy barrier in the rate-determining step.^59^ Pd_1_/ZnO nanowires also achieved full CO conversion, though prolonged use led to agglomeration, highlighting the need for robust structural stabilization in SAC systems.^60^ In addition to oxidation, SACs are being explored for electrochemical CO reduction into valuable chemicals like methanol (CH_3_OH).^61^

A comparative study on NiO–Al_2_O_3_ catalysts for CO_2_ methanation confirmed mechanochemistry as the most effective synthesis method. The ball-milled catalyst with 20 wt% NiO exhibited the highest CO_2_ conversion (68%), CH_4_ selectivity (96%), and superior thermal stability due to its high surface area and small crystal size.^62^ The solvent-free nature of the method and the ability to enhance NiO–Al_2_O_3_ interaction were key factors in its superior performance over traditional techniques.^63^ The diverse range of mechanochemically synthesized catalysts for CO_2_ hydrogenation demonstrates the technique's versatility. However, several cross-cutting challenges emerge: (1) ball milling parameters are interdependent and difficult to standardize, leading to variability in catalyst properties across different laboratories and equipment types. (2) While solvent-free, the electrical energy consumed during prolonged high-energy milling must be factored into overall process sustainability, especially for industrial-scale production. (3) Most studies report performance under idealized lab conditions. Real industrial CO_2_ streams contain impurities (H_2_S, NO_*x*_, particulates) that may deactivate catalysts through poisoning or physical blockage of active sites. (4) The cost of noble metals (Pd, Pt, Rh) in SACs and the infrastructure investment for industrial ball mills must be weighed against performance benefits.

### 2.2 Compounds with imidazole-silane fragment for cyclic carbonate synthesis

**Fig. 3:**
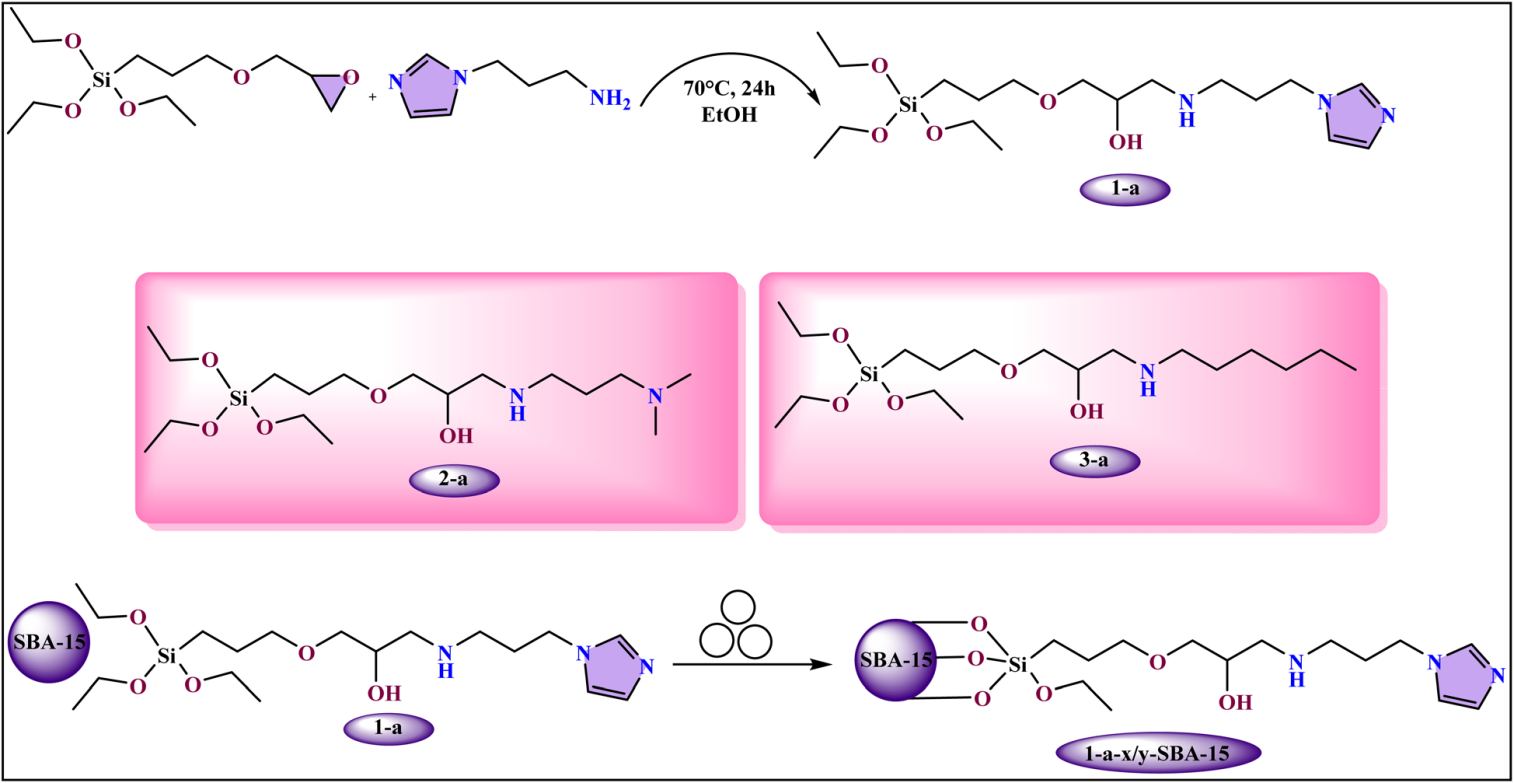
Schematic illustration of catalyst immobilization on SBA-15, along with the structural depiction of silane compound *n*-a.

Mechanochemistry is being applied to the synthesis of compounds with imidazole-silane fragment, moving beyond traditional metal-based systems. A new method has been developed for synthesizing mesoporous supported compounds, SiO_2_ with alkylolamine sites for CO_2_ cycloaddition reactions.^64^ This approach, which is entirely solvent-free and rapid, enables the covalent grafting of compounds with imidazole-silane fragment onto a silica support *via* ball milling within just 15 minutes.^65^ The synthesis is a two-step process (Fig. 3). First, a compound (*n*-a) is formed through a ring-opening reaction between a primary amine and an epoxy compound.^66^ In the second step, new compound with imidazole-silane fragment is grafted onto mesoporous silica (SBA-15) using a planetary mill. A mixture of the silica support and the catalyst was subjected to ball milling at high speeds for a short period. The final catalyst (1-*a*-60/*b*-SBA-15) exhibits high efficiency in converting CO_2_ and epoxides into cyclic carbonates without the need for halogen cocatalysts. It also demonstrates excellent stability and reusability, a significant advantage over conventional methods that often require harsh conditions and solvents. The effectiveness of this mechanochemical approach has been confirmed through both experimental and theoretical studies, highlighting its sustainability and potential for greener, scalable catalyst production.^67^

**Fig. 4:**
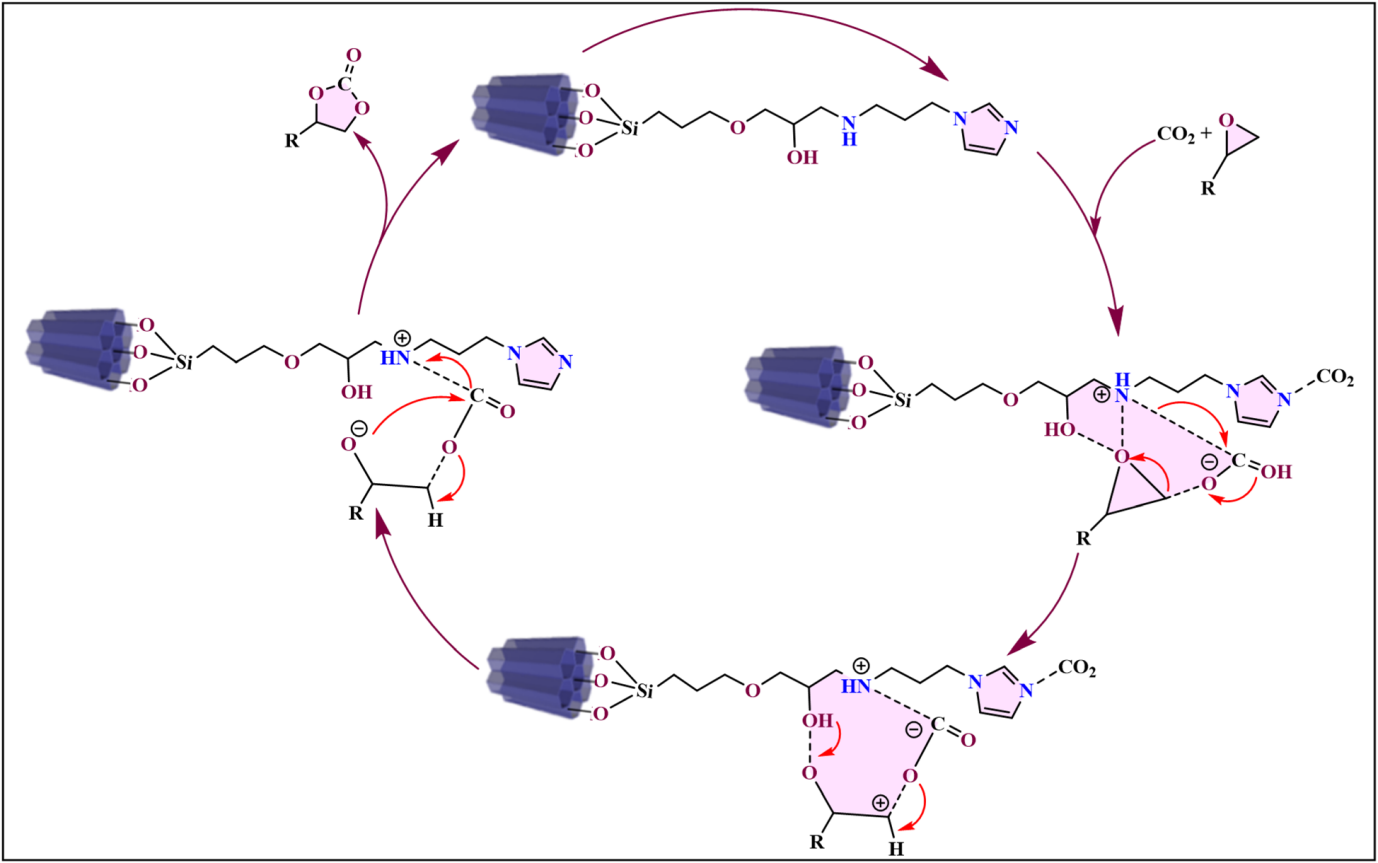
Schematic representation of the proposed catalytic pathway for cyclic carbonate synthesis using 1-a supported on SBA-15.

A proposed mechanism for this reaction highlights the collaborative role of the catalyst's functional groups.^68^ The –OH and N–H groups act as hydrogen bond donors to activate the epoxide, while the nitrogen of the imidazole group serves as a Lewis base to activate CO_2_. Subsequently, a nucleophilic attack by the activated CO_2_ on the epoxide leads to a carbonate half-ester intermediate, which then cyclizes to form the final cyclic carbonate (Fig. 4).^69^ This approach offers a greener and more scalable route to catalyst production compared to conventional methods.

While these catalysts avoid issues related to metal leaching and toxicity, their activity is generally lower than metal-based systems, requiring higher catalyst loadings or longer reaction times. The grafting efficiency during ball milling can be incomplete, leading to a mixture of bound and physiosorbed catalytic species, which may leach during recycling. Additionally, the harsh mechanical forces may damage the delicate mesoporous structure of SBA-15, reducing surface area and pore accessibility over multiple milling cycles.

### 2.3 Photocatalysts for CO_2_ reduction

Photocatalytic CO_2_ reduction (CO_2_RR) has emerged as a sustainable approach to convert CO_2_ and H_2_O into valuable fuels such as CO, CH_4_, and small hydrocarbons using solar energy. The performance of these reactions is highly dependent on the nature of the catalyst, which must efficiently harvest light, separate charge carriers, and activate CO_2_ molecules.^70^ Mechanochemistry has recently enabled the design of advanced photocatalytic materials, including single-atom catalysts (SACs) and perovskite-based systems, which provide well-defined active sites, enhanced electron–hole separation, and improved CO_2_ adsorption. SACs supported on conductive matrices like g-C_3_N_4_ or carbon frameworks exhibit high atomic efficiency and selectivity due to their tunable coordination environment and strong interaction with supports, facilitating CO_2_ activation and selective product formation {Haider, 2024 #487}{Qureshi, 2023 #488}. In parallel, defect-rich metal oxides, heterojunction composites, and doped semiconductor photocatalysts enhance light absorption and prolong charge carrier lifetimes, further improving photocatalytic efficiency. Collectively, these developments illustrate a trend toward rational design of photocatalysts combining atomic-level active sites, engineered defects, and optimized interfaces, pushing CO_2_ photoreduction closer to practical solar-to-fuel applications.

#### 2.3.1 Thianthrene-based donor–acceptor ladder polymers

**Fig. 5:**
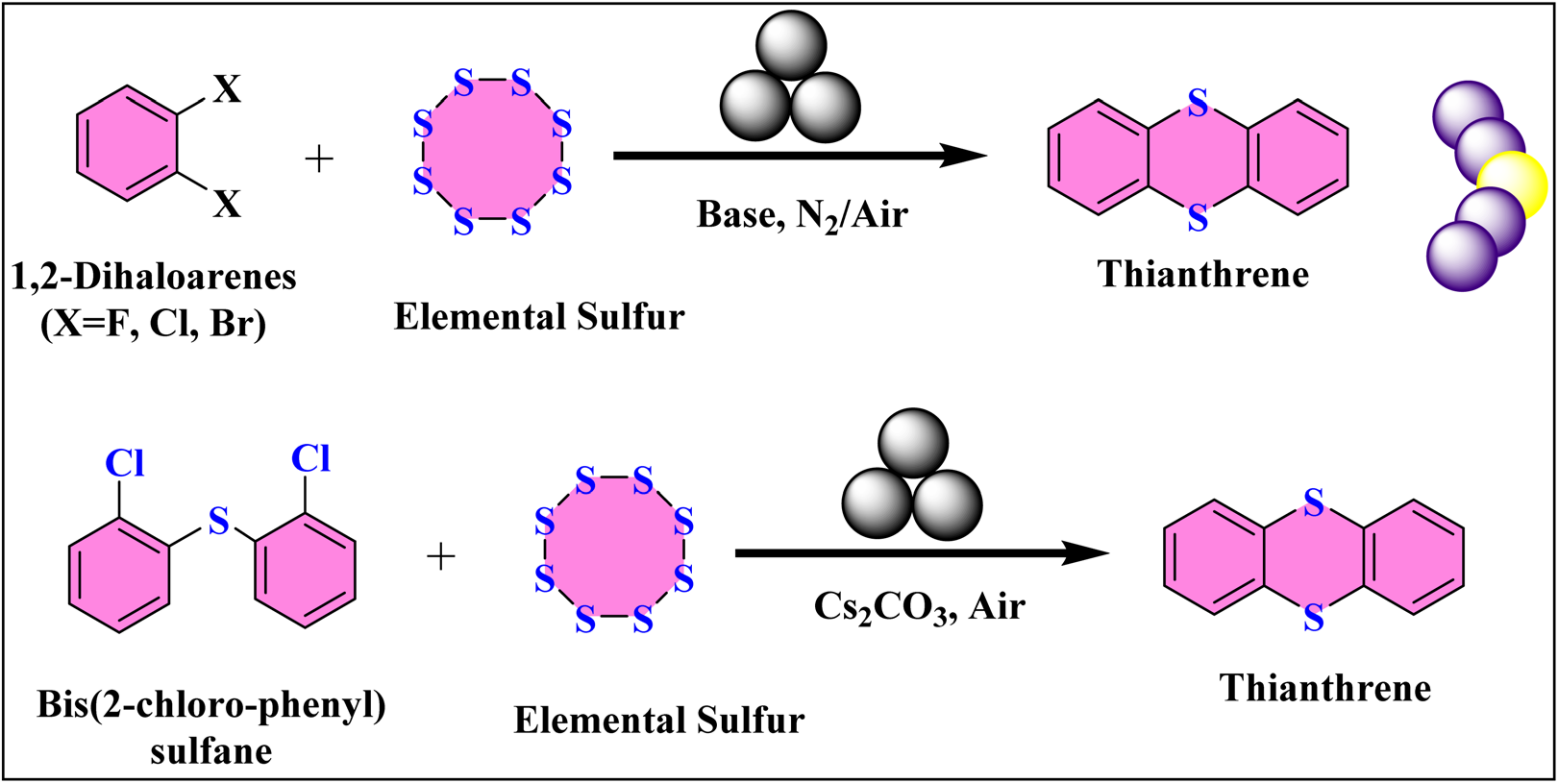
Mechanochemical nucleophilic aromatic substitution (SNAr) synthesis of thianthrene from elemental sulfur and 1,2-dihaloarenes.

A recent study has successfully used a mechanochemical strategy to transform elemental sulfur (S_8_), a common byproduct of petroleum refining, into a thianthrene-bridged donor–acceptor (D–A) porous ladder polymer.^71^ This was achieved through a solid-state nucleophilic aromatic substitution (SNAr) reaction between sulfur and 1,2-dihaloarenes during ball milling.^72^ To construct the thianthrene ring, a novel solid-state condensation reaction was initiated by combining 1,2-dichlorobenzene with elemental sulfur in a ZrO_2_ milling jar (Fig. 5).^73^ Using Cs_2_CO_3_ as a base and ball milling at 30 Hz for 5 hours, the desired thianthrene compound was obtained with a yield of approximately 51%. The method proved remarkably robust, with a comparable yield of 54% even when performed under ambient air instead of a nitrogen atmosphere. The reaction pathway was further confirmed by using a sulfur-linked model molecule, bis(2-chlorophenyl) sulfane, which yielded thianthrene with an even higher yield of ∼69% under similar conditions.^74^ This highlights the effectiveness of mechanochemical SNAr for creating these bent thianthrene units. The broad applicability of the method was demonstrated by its success with other 1,2-dihaloarenes, such as 1,2-fluorobenzene and 1,2-dibromobenzene, which are also reactive.^75^ The method is robust, rapid, and broadly applicable to various dihaloarenes, demonstrating a new, sustainable pathway to both utilize waste sulfur and create highly effective photocatalysts for CO_2_ reduction.

**Fig. 6:**
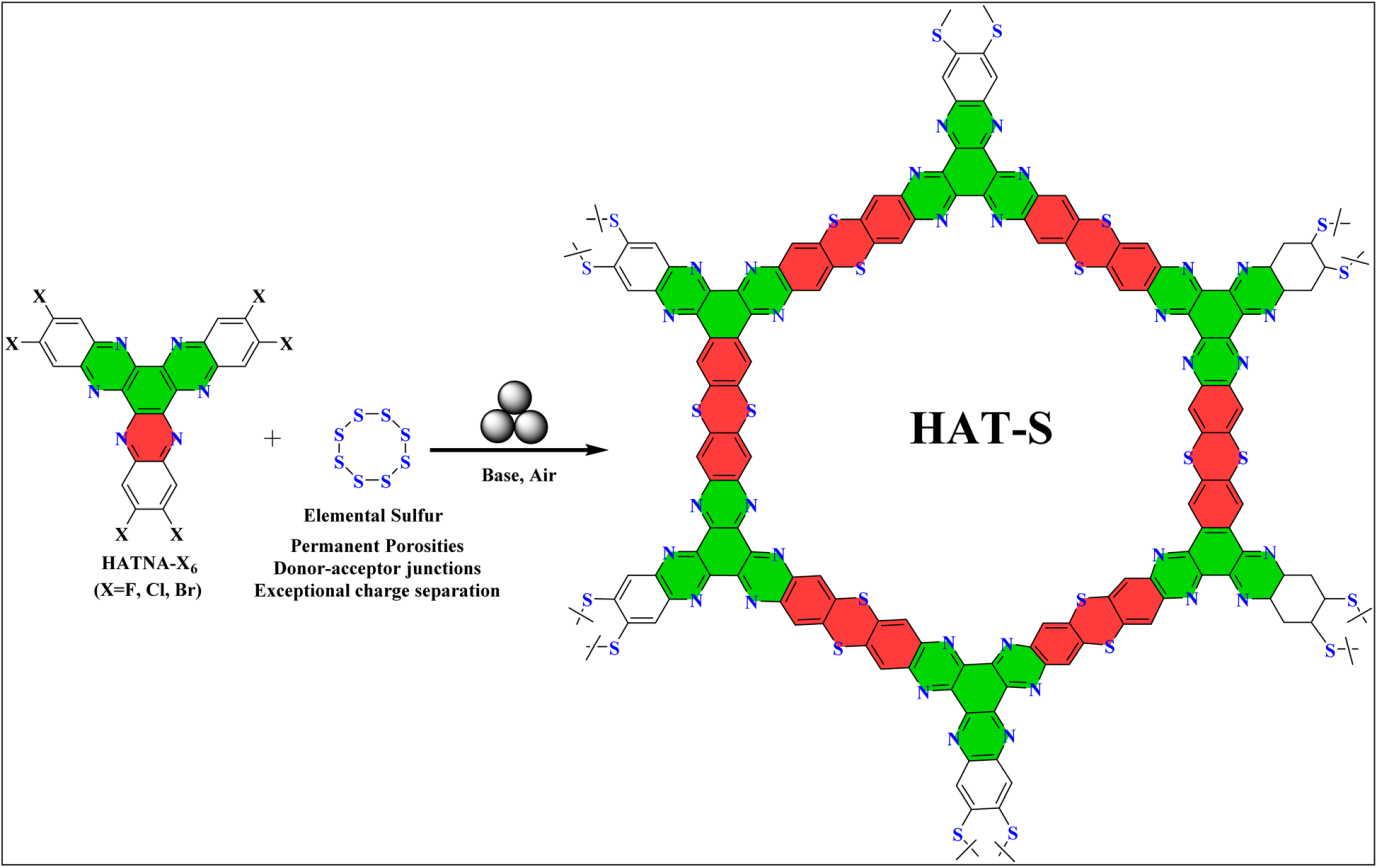
Mechanochemical SNAr-based synthesis of thianthrene-bridged donor–acceptor (D–A) porous ladder polymer networks.

Building on this, a highly advanced thianthrene-bridged donor–acceptor (D–A) ladder polymer networks was developed using 2,3,8,9,14,15-hexachloro-5,6,11,12,17,18-hexaazatrinaphthylene (HATNA-Cl_6_) as a *C*_3_-symmetric electron-deficient monomer (Fig. 6).^76^ The process involved milling the HATNA-Cl_6_ monomer with S_8_ and Cs_2_CO_3_ in a ZrO_2_ jar for 2 hours. This mechanochemical approach yielded a novel class of thianthrene-bridged porous ladder polymers featuring dense D–A junctions, which functioned as an effective photocatalyst for CO_2_ reduction. The material achieved an impressive CO production rate of 306.1 µmol g^−1^ h^−1^ with nearly 100% CO selectivity, using only water vapor as an electron donor and visible light irradiation. This remarkable performance, without external photosensitizers, sacrificial agents, or cocatalysts, underscores the potential of mechanochemistry in creating cutting-edge materials for sustainable photocatalysis.^77^

#### 2.3.2 Metal halide perovskite photocatalysts

**Fig. 7:**
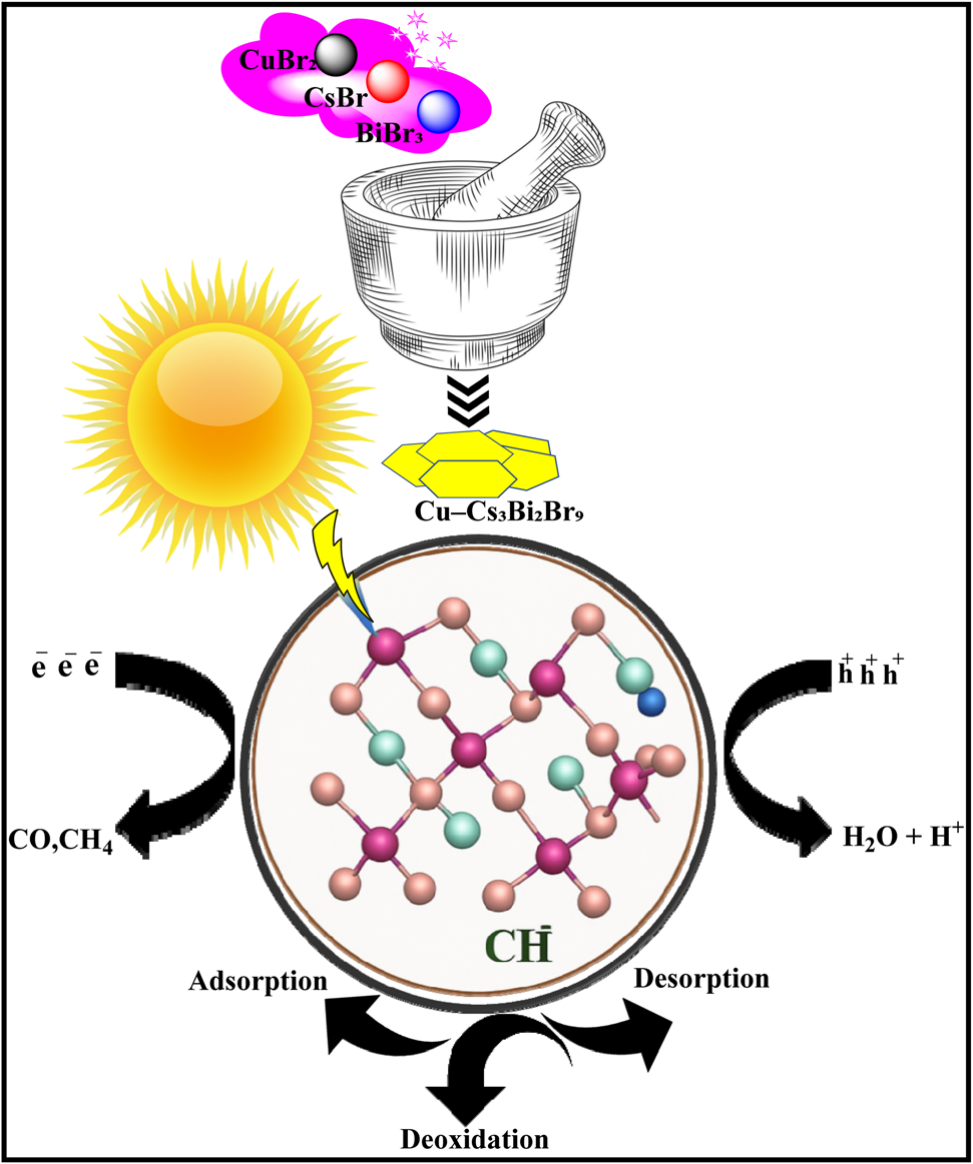
Schematic diagram illustrating the mechanochemical reaction pathways involved in CO_2_ photoreduction.

Similarly, mechanochemistry has been used to synthesize a bismuth-based metal halide perovskite, Cs_3_Bi_2_Br_9_ (CBB), which is known for its favorable photophysical properties but suffers from poor selectivity and carrier recombination.^70^ A green, one-step mechanochemical method was developed to create Cu-doped ultrathin 2D CBB nanoplates. This process avoids the high temperatures and organic solvents of traditional methods and directly enhances the catalyst's performance. The optimized Cu-doped CBB nanoplates achieved a CO generation rate of 100.04 µmol g^−1^ h^−1^ (4.25 times higher than pristine CBB) with a selectivity of 98.60%.^78^ The improved performance is due to the high surface area of ultrathin structure and the synergistic effect of Cu doping, which enhances charge transfer and lowers the energy barrier for key reaction intermediates (Fig. 7).^79^

#### 2.3.3 Halide perovskites with conductive supports

One area of notable advancement is the synthesis of halide perovskites such as CsPbBr_3_ through mechanochemistry.^80^ This method allows for precise control over nanostructure shapes, including nanorods, nanospheres, and nanosheets, which significantly influences photocatalytic behavior. Further enhancements have been achieved by integrating conductive supports like reduced graphene oxide (RGO) and co-catalysts such as Cu nanoparticles. For example, incorporating Cu-RGO into CsPbBr_3_ nanosheets led to a tenfold increase in methane selectivity and significantly improved stability, retaining over 90% performance across multiple cycles. This highlights how mechanochemical hybridization with functional nanomaterials can overcome the common drawbacks of pristine perovskites, such as poor charge separation and instability under humid conditions.^80^ This approach also aligns with circular economy principles by avoiding hazardous reagents and reducing energy consumption. By offering control over structure–function relationships and fostering the creation of robust composite systems, mechanochemical strategies are well-positioned to advance the field of photocatalytic CO_2_ reduction, supporting the design of high performance solar-to-fuel systems.^81^ The approach of integrating conductive supports like reduced graphene oxide (RGO) and co-catalysts such as Cu nanoparticles has also been explored to further enhance photocatalytic efficiency and stability, overcoming common drawbacks of pristine perovskites.^82^

Photocatalytic CO_2_ reduction offers the advantage of utilizing solar energy directly, but current efficiencies remain far below what is required for industrial application.^83^ The reported activities (typically < 500 µmol g^−1^ h^−1^) translate to very low quantum efficiencies (often <1%), indicating that most incident photons are not effectively utilized.^83*b*^ Moreover, mechanochemically synthesized photocatalysts face several inherent challenges. (1) Halide perovskites, despite enhanced stability through Cu–RGO compositing, still undergo degradation under prolonged UV irradiation and humid conditions.^84^ In addition, the mechanical stress induced during high-energy ball milling may introduce structural defects that act as charge recombination centers, partially offsetting the gains in photocatalytic activity.^85^

(2) While donor–acceptor (D–A) ladder polymers exhibit excellent CO selectivity, their practical scalability remains a concern due to synthetic complexity and cost.^77^ Donor–acceptor (D–A) ladder polymers used in mechanochemical photocatalysis are made up of alternating electron-rich donor and electron-deficient acceptor units that are covalently bonded together to form a rigid, planar ladder-type backbone.^86^ This topology reduces torsional disturbance and encourages extended π-electron delocalization, which is necessary for effective charge separation and transport.^87^ HATNA-Cl_6_ (hexachloro-hexaazatrinaphthylene), a typical acceptor unit, is a completely fused polycyclic aromatic framework with six nitrogen atoms.^88^ Nitrogen heteroatoms and chlorine substituents reduce the LUMO energy level, allowing for efficient electron transfer from donor segments during photoexcitation and stabilizing charge-separated states that promote selective CO production during CO_2_ reduction.^89^ The donor units, which are typically electron-rich aromatic or heteroaromatic moieties, increase visible-light absorption and allow photogenerated electron supply.^89*b*^ When produced mechanochemically, these components create stiff, conjugated networks with fewer non-radiative recombination paths.^90^ HATNA-Cl_6_ synthesis includes many halogenation and condensation processes, necessitating expensive precursors and extended milling durations (2–5 h), which may limit economic feasibility for large-scale applications.^91^

(3) Porous photocatalysts require gas–solid interaction, but particle aggregation during milling might clog pore channels, lowering available surface area for CO_2_ adsorption and light harvesting.^92^ (4) Finally, many photocatalytic investigations lack strict controls for carbon contamination. Organic leftovers from manufacturing or handling may degrade under UV irradiation, increasing the apparent CO_2_ reduction rate. Isotope-labeling experiments with ^13^CO_2_ are critical for confirming real catalytic activity, but remain underutilized.^93*d*^

### 2.4 Catalysts for biogas reforming and syngas production

Expanding the scope of mechanochemistry further, this technique is proving valuable in the catalytic bi-reforming of biogas into syngas.^94^ Biogas, a mixture of methane and CO_2_, is a sustainable feedstock for producing syngas with an ideal H_2_/CO ratio of ∼2 for downstream processes like methanol synthesis.

#### 2.4.1 Ruthenium catalysts for Bi-reforming

**Fig. 8:**
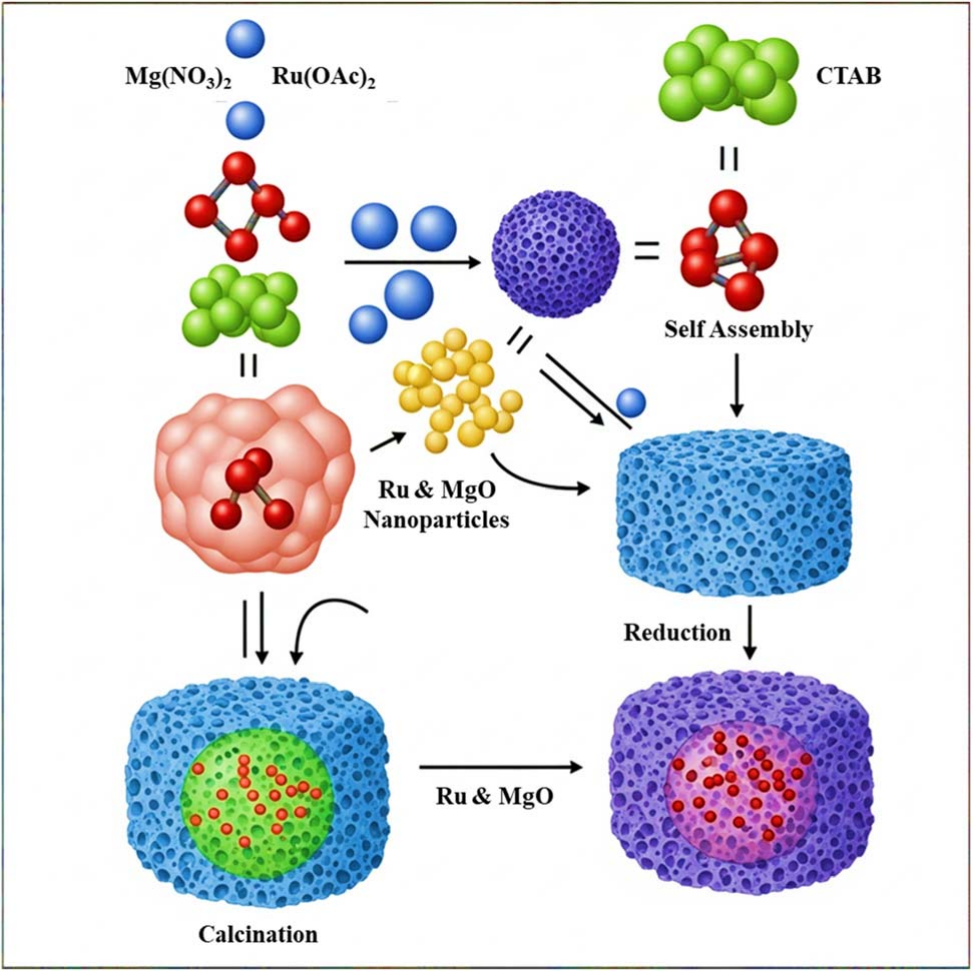
Schematic illustration of the synthesis route for Ru-loaded MgO using CTAB as a structure-directing agent.

Ruthenium-based catalysts are highly effective for this reaction due to their coking resistance. A highly efficient catalyst, 0.2Ru/MgO-0.2CTAB (cetyltrimethylammonium bromide), was synthesized using a soft template-assisted mechanochemical technique. This method, which involves ball milling with the CTAB template, produces ultrafine Ru nanoparticles (∼1.1 nm) homogeneously dispersed on a porous MgO support. The resulting material achieved high CO_2_ conversion (61%) and CH_4_ conversion (∼94%), maintaining its performance for over 120 hours (Fig. 8).^95^ This superior stability is a direct result of the mechanochemically optimized structure, which creates strong metal-support interactions that prevent sintering and a porous support that enhances CO_2_ activation and gas diffusion.^96^

#### 2.4.2 Fischer–Tropsch hydrotalcite catalysts

The global shift toward sustainable energy sources has driven interest in the Fischer–Tropsch synthesis (FTS), a process that converts syngas into a range of hydrocarbons.^97^ While traditional FT catalysts are often Fe, Co, and Ru-based, new synthesis methods are being explored to improve performance. Hydrotalcites (HTs), for example, are a class of materials that can be synthesized using co-precipitation, ultrasound, or ball milling techniques.^98^ A study comparing Fe-based HT catalysts for FT synthesis found that while both ultrasound and ball-milling-prepared catalysts achieved high CO and CO_2_ conversions (∼98.5%) at 300 °C, their product selectivity differed significantly. The ultrasound-processed catalyst favored longer-chain hydrocarbons (C_7_^+^) while the ball milling prepared catalyst was more selective for shorter-chain products (C_2_–C_6_). This further demonstrates how the synthesis method directly impacts the final catalyst structure and its performance.^99^

While mechanochemically synthesized Ru/MgO shows impressive performance, several practical issues limit immediate deployment: (1) ruthenium, though more abundant than Pt or Rh, remains expensive. For large-scale biogas upgrading facilities, catalyst cost per ton of syngas produced becomes a critical economic factor. Development of Ni-based alternatives with similar coking resistance is necessary. (2) Real biogas composition varies significantly depending on source (agricultural waste, municipal sewage, landfill gas), containing not just CH_4_ and CO_2_ but also H_2_S, NH_3_, siloxanes, and moisture. These impurities can poison the catalyst or cause equipment corrosion. Pre-treatment requirements add cost and complexity. (3) Both dry reforming (CO_2_ + CH_4_) and steam reforming components of bi-reforming are highly endothermic. Efficient heat integration and reactor design are critical for maintaining catalyst bed temperature uniformity, especially given the small Ru particle size which may be more prone to thermal gradients. (4) Despite Ru's inherent coking resistance, operation with biogas typically requires periodic regeneration. The mechanochemically created porous MgO structure may undergo gradual densification during oxidative regeneration cycles, reducing its long-term effectiveness.

### 2.5 Dual-functional materials for integrated CO_2_ capture and conversion

Another innovative application of mechanochemistry is in integrated CO_2_ capture and utilization (ICCU), where CO_2_ adsorption and catalytic conversion are deliberately combined within a single material platform. In this context, dual-functional materials (DFMs) are specifically designed to possess two distinct but complementary types of active sites: acid–base sites responsible for CO_2_ capture and redox-active sites that catalyze subsequent CO_2_ conversion. Mechanochemistry has been successfully employed to synthesize such DFMs from natural minerals, particularly CaO and Fe, enabling intimate contact between these heterogeneous functionalities.^100^ During solvent-free ball milling, enhanced Fe–Ca interfacial bonding is generated, promoting spatial proximity between basic CaO adsorption sites and redox-active Fe centers. As a result, the mechanochemically prepared DFM exhibited a high CO_2_ capture capacity of 5.5 mmol g^−1^ and a CO_2_ conversion efficiency of 87%, with 100% selectivity toward CO, significantly outperforming catalysts prepared without mechanochemical treatment.^100^ The addition of MgO further improved durability by acting as a thermal stabilizer and sintering inhibitor, limiting CO_2_ capture loss to <15% after 20 cycles. Beyond performance enhancement, the mechanochemical route also demonstrated clear techno-economic advantages, with a projected ∼20% reduction in operational costs compared to conventional ICCU processes.^101^

**Fig. 9:**
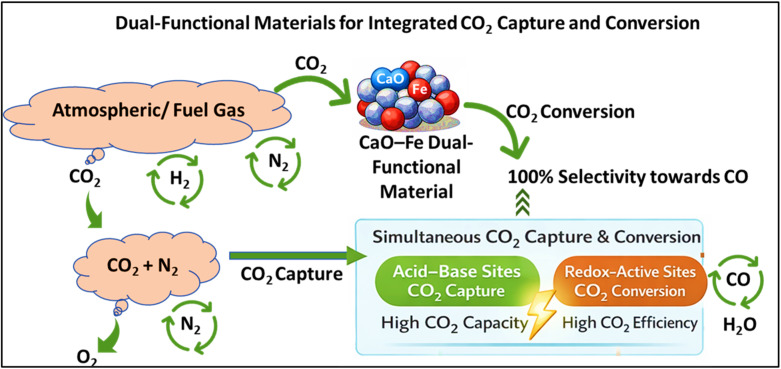
Schematic illustration of dual-functional materials (DFMs) for integrated CO_2_ capture and conversion.

The mechanochemical synthesis of RuNi-based bimetallic DFMs supported on CeO_2_–Al_2_O_3_ represents another important advancement in ICCU materials design.^102^ In these systems, CaO-derived basic sites facilitate CO_2_ adsorption, while RuNi bimetallic redox sites catalyze CO_2_ hydrogenation through the reverse water–gas shift (RWGS) reaction, demonstrating the general applicability of the acid–base/redox dual-site concept. Dry co-milling ensures uniform dispersion of the RuNi active phase and minimizes the spatial separation between capture and conversion sites, thereby enhancing overall ICCU performance. Although premature CO formation was initially observed, this challenge was effectively mitigated by introducing O_2_ into the feed, enabling stable operation under flue-gas-like conditions. Notably, these DFMs also demonstrated direct air capture (DAC) capability, highlighting their potential relevance for practical carbon management applications (Fig. 9).^103^

Despite the promise of DFM-based ICCU systems, several challenges must be addressed before industrial implementation. Repeated temperature swings between CO_2_ capture and conversion can induce sintering or phase changes, leading to gradual capacity loss even in stabilized systems. In addition, the mismatch between slow CO_2_ adsorption kinetics and faster catalytic conversion complicates reactor design and process integration. The reliance on hydrogen as a reductant further raises concerns regarding overall carbon footprint and cost unless renewable H_2_ sources are employed. Finally, although direct air capture has been demonstrated, the low atmospheric CO_2_ concentration imposes significant energy penalties, underscoring the need for further improvements in sorbent capacity, selectivity, and process efficiency.

### 2.6 Mechanochemical routes for clean hydrogen generation

Mechanochemical strategies are also being developed for clean hydrogen generation, aiming to produce H_2_ without contaminating CO or other carbon byproducts. A novel process uses high-energy ball milling to drive room temperature CO_2_ hydrogen desorption reactions with alkali hydrides (LiH, NaH, KH).^104^ In this process, alkali hydride powders react with CO_2_ under mechanical stress to produce pure hydrogen gas. NaH and KH were found to be particularly effective, yielding hydrogen mole fractions as high as 98.72% without any CO_*x*_ byproducts.^105^ The reaction converts CO_2_ to solid carbon and alkali carbonates, ensuring complete CO_2_ utilization and eliminating harmful emissions. The efficiency of this process is highly dependent on milling speed and reaction duration, with KH showing the highest efficiency. This solvent-free, room-temperature method provides a cost-effective and environmentally sound route for producing clean hydrogen, aligning with global clean energy goals.^106^

### 2.7 Additional mechanochemical applications

#### 2.7.1 Direct CO_2_ conversion to elemental carbon

Beyond fuels and chemicals, a truly sustainable approach is the direct conversion of CO_2_ into elemental carbon, a valuable material for applications in energy storage and catalysis.^107^ Conventional methods for producing carbon from precursors like biomass often release more CO_2_.^108^ While metal carbonates can be used in high-temperature electrolytic processes, these methods are energy-intensive.^109^ Mechanochemical ball milling, however, has emerged as a promising, low-energy alternative. This method can synthesize porous carbon from CO_2_ and sodium hydride at room temperature in as little as one minute.^110^ The morphology and porosity of the resulting carbon are highly dependent on the milling conditions. The process is activated by mechanical energy, and the resulting hierarchical meso and macroporous carbon structures are formed by a “gas blowing effect” and self-templating by Na_2_CO_3_.^111^ This green and scalable method represents a significant step toward efficiently transforming CO_2_ into high-value carbon materials.^110^

#### 2.7.2 Electrochemical CO_2_ reduction catalysts

For the electrochemical reduction of CO_2_ to CO, mechanochemistry has been successfully applied to produce defect-rich CuO-based composite catalysts. The dry milling of CuO with Sn and In resulted in a Sn–In–CuO catalyst that demonstrated a remarkable 96.11% faradaic efficiency for CO production. The mechanochemical process introduced defects and enhanced electronic conductivity, leading to a high electrochemically active surface area and improved CO_2_ adsorption kinetics. This work highlights mechanochemistry as a green and scalable route for tailoring catalyst structure and performance for selective CO generation.^112^ The details of mechanochemically synthesized catalysts and their promoters employed in CO_2_ reduction are summarized in Tables 1 and 2.

**Table 1:** Main types of mechanochemically synthesized catalysts and their CO_2_ conversion applications

Catalyst type	Metal composition	Reaction type	Key products	Operating conditions	Performance metrics	Key advantages	Representative examples	Synthetic challenges	Ref.
Iron-based catalysts	Fe, Fe–Mg–K, Fe–Cu	Fischer–Tropsch synthesis (FTS)	C_2_–C_4_ olefins, hydrocarbons	200–350 °C, 1–5 MPa	CO_2_ conv. 32.1%; olefin sel. 55.4%	Enhanced chain-growth control, suppressed methane formation	Fe–Mg–K, Na–Fe–Cu	Uniform dispersion of promoters, phase control, prevention of sintering during high-temperature activation	24
Nickel-based catalysts	Ni, Ni–MgO, Ni/Al_2_O_3_, Ni/Cr_2_O_3_	CO_2_ methanation (Sabatier)	CH_4_	300–400 °C, 0.5–1.0 MPa	CO_2_ conv. 75.2–85.2%; CH_4_ sel. 96–99.5%	High activity, thermal stability, industrial scalability	Ni–MgO/MgH_2_, Ni/Al_2_O_3_	Avoiding Ni sintering, controlling particle size, metal-support interaction optimization	31*b*
Bimetallic catalysts	Ni–Co, Ni–Cu, Ru–Ni	Methanation, RWGS	CH_4_, CO	300–350 °C, 1.0 MPa	CO_2_ conv. 84.5%; CH_4_ sel. 99.8%	Synergistic effects, enhanced electron transfer	Ni_2_Co_3_/SiO_2_, RuNi/CeO_2_–Al_2_O_3_	Achieving homogeneous alloying, preventing phase segregation, precise composition control	15*c*
Copper-based systems	Cu, Cu–Zn, Cu–Zr	Methanol synthesis, CO reduction	CH_3_OH	200–300 °C, 5–10 MPa	High methanol yield	Superior selectivity, cost-effectiveness	Cu–ZrO_2_, CuSA/TiO_2_	Stabilizing Cu oxidation states, preventing nanoparticle agglomeration, reproducible impregnation	41*b*
Ruthenium catalysts	Ru, Ru/MgO	Biogas bi-reforming	Syngas (CO + H_2_)	600–700 °C, 1.0 MPa	CO_2_ conv. 61%; CH_4_ conv. 94%	Excellent coking resistance, long-term stability	0.2Ru/MgO–CTAB	High Ru cost, controlled dispersion on support, sintering prevention at high temperatures	99*b*
Manganese oxide catalysts	MnO_*x*_, Pt–MnO_*x*_, Cu–MnO_*x*_	CO_2_ hydrogenation	CH_4_, alcohols	550–850 °C	Activity enhanced 12–13× with doping	Tunable oxidation states, low cost	MnO_*x*_ (600 rpm milling)	Controlling Mn oxidation states, reproducible doping, phase stability at high temperature	110
Perovskite materials	Cs_3_Bi_2_Br_9_, CsPbBr_3_, Cu-doped CBB	Photocatalytic CO_2_ reduction	CO, CH_4_	Room temperature, visible light	CO yield 100–306 µmol g^−1^ h^−1^; sel. 98–100%	Solar-driven, cocatalyst-free	Cu-doped CBB, CsPbBr_3_–Cu–rGO	Achieving uniform doping, controlling defect sites, moisture/air sensitivity	69
Single-atom catalysts (SACs)	Pd-SA, Cu-SA, Rh-SA, Pt-SA	Photoreduction, CO_2_RR	CO, CH_4_, syngas	RT–100 °C, visible/UV	CO_2_RR rate 271.6 µmol g^−1^ h^−1^ (Pd/TiO_2_)	Atomic dispersion, maximal atom efficiency	PdSA/TiO_2_, CuSA/ZnO	Stabilizing single atoms, avoiding clustering, scalable synthesis	
Multi-metallic catalysts	K–Cu–Fe–ZnO–Al_2_O_3_ (KCFZA)	Alcohol synthesis	Methanol, ethanol, acetic acid	250–300 °C, 5 MPa	Tunable alcohol selectivity	Milling-controlled product distribution	K–Cu–Fe/ZnO–Al_2_O_3_	Homogeneous element distribution, complex multi-metal synergy, reproducibility	77
Hydrotalcite-based catalysts	Fe-HT, MgCuFe-HT	Fischer–Tropsch synthesis	C_2_–C_6_, C_7_^+^ hydrocarbons	∼300 °C	CO + CO_2_ conv. ∼98.5%	Composition-controlled selectivity	Fe-based HT, MgCuFe-BM	Controlling layer stacking, uniform cation distribution, prevention of dehydroxylation	2
Organic/hybrid catalysts	Imidazole-silane/SBA-15, D–A polymers	Cyclic carbonate synthesis, photocatalysis	Cyclic carbonates, CO	80–120 °C; visible light	∼100% conversion and selectivity	Solvent-free, reusable, halogen-free	1-*a*-60/*b*-SBA-15, thianthrene D–A	Achieving high surface area, functional group stability, reproducible polymerization	
Dual-functional materials (DFMs)	CaO–Fe, RuNi/CeO_2_–Al_2_O_3_–CaO	ICCU (capture + conversion)	CO, CH_4_	500–700 °C, 1.0 MPa	CO_2_ cap. 5.5 mmol g^−1^; conv. 87%	Integrated sorption-conversion, flue-gas compatible	CaO–Fe, RuNi/CeO_2_–Al_2_O_3_	Balancing sorption and catalytic sites, preventing sintering, maintaining mechanical stability	79

**Table 2:** Key promoters and support materials in mechanochemical catalyst design

Support material	Promoter elements	Primary function	Effect on performance	Underlying mechanism	Ref.
Al_2_O_3_	Mn, La, Ni	Metal dispersion, reducibility	↑ Surface area; ↑ MSI	Lewis's acid–base interactions, oxygen vacancies	43, 62*a* and 96
MgO	Mn, La, Cr, Ca	Thermal stability, CO_2_ activation	↑ Durability; ↑ CO_2_ chemisorption	Strong basic sites, enhanced MSI	62*b*, 101 and 103
SiO_2_	Ni, Co, Pt, Cu	Metal anchoring	Uniform active-site distribution	Silanol–metal interactions	52 and 106*a,c*
ZrO_2_	Cu, Ni	Redox activity, H_2_ dissociation	↑ Defect density; ↑ activity	Oxygen vacancies, Lewis's acidity	49 and 113
CeO_2_	Ru, Ni, Cu	Oxygen storage, electron transfer	Enhanced RWGS; ↑ CO selectivity	Ce^3+^/Ce^4+^ redox cycling	53, 57 and 81
Cr_2_O_3_	Mn, La, Fe	Metal stabilization, coke suppression	↑ Long-term stability	Strengthened metal–support interaction	51 and 112

### 2.8 Challenges and limitations of mechanochemical catalyst synthesis

While the preceding sections have highlighted numerous successes, a comprehensive evaluation requires critical examination of the inherent limitations and unresolved challenges in mechanochemical catalyst synthesis for CO_2_ reduction.

#### 2.8.1 Reproducibility and standardization issues

Ball milling equipment varies significantly across manufacturers and laboratories. Planetary mills, shaker mills, and vibratory mills impart different mechanical energy profiles. Even within the same mill type, factors like jar material (steel, ZrO_2_, tungsten carbide), ball size distribution, ball-to-powder ratio, filling degree, and rotation/vibration frequency create a multi-dimensional parameter space that is difficult to standardize.^114^ A “30 Hz, 2 hour” milling protocol in one lab may produce vastly different results in another due to these equipment differences. Unlike solution chemistry where reaction kinetics can be modeled based on concentration, temperature, and activation energy, the “chemistry by collision” in ball milling is stochastic and difficult to predict.^115^ Local temperatures during ball-powder collisions may transiently reach 1000+ °C, but for only microseconds. The resulting material is an ensemble average of countless collision events with varying energy transfer, making rational design challenging. Conventional *ex situ* characterization (XRD, TEM, XPS) provides snapshots of the final product but reveals little about the mechanochemical process itself. *Operando* techniques for ball milling are in early stages of development, leaving a black box between input materials and output catalysts.^116^

#### 2.8.2 Scalability and energy economics

High-energy ball milling consumes significant electrical energy (typically 0.1–1 kW h per gram of material for extended milling). For catalysts to be used in energy applications (fuel production), a critical question is whether the energy return on energy invested (EROEI) is favorable.^117^ If producing 1 kg of mechanochemical catalyst requires 500 kW h, and that catalyst generates fuel equivalent to 400 kW h over its lifetime, the net energy balance is negative. Laboratory ball mills typically process 10–100 g per batch. Industrial-scale catalyst production requires tons per day.^118^ While industrial ball mills exist (used in mining and cement industries), they operate at much lower specific energy inputs than the high-energy research mills used in published studies.^119^ The catalytic properties achieved in lab-scale synthesis may not translate to industrial milling conditions optimized for throughput rather than product quality. Prolonged milling causes wear of balls and jar walls, introducing contaminant metals (Fe, Cr, W) into the product. For some catalysts, this contamination may be beneficial (inadvertent doping), but for others it can poison active sites. The need for frequent replacement of milling media adds to operational costs and generates waste.^120^

#### 2.8.3 Long-term stability under industrial conditions

The high defect densities that enhance initial activity are often metastable. Under reaction conditions (elevated temperature, pressure, reactive atmosphere), defects can anneal out, grain boundaries migrate, and nanostructures coarsen.^121^ Many studies report excellent performance over 10–100 hours, but industrial catalysts must function for years (10 000–50,000 hours). Industrial CO_2_ sources (flue gas, cement plants, steel mills) contain sulfur compounds, nitrogen oxides, particulates, and trace heavy metals.^122^ Mechanochemically synthesized catalysts with their high surface areas and open structures may be more vulnerable to poisoning and fouling than robust conventional catalysts designed for harsh environments.^123^ When catalysts deactivate due to coking, sintering, or poisoning, regeneration (typically oxidative burning or chemical treatment) may destroy the carefully engineered nanostructures created by mechanochemistry.^124^ If a catalyst requires regeneration every few months and properties cannot be fully restored, the economic advantage over disposable catalysts diminishes.^125^

#### 2.8.4 Product selectivity control

Mechanochemical synthesis often creates a heterogeneous distribution of active sites (corners, edges, defects, terraces) with varying activities and selectivities. While this diversity can be beneficial (*e.g.*, Ni–Co bimetallic catalysts), it can also lead to unwanted side reactions and product mixtures.^126^ Fine-tuning selectivity requires understanding which sites are responsible for which products knowledge that is currently limited. Many mechanochemically synthesized catalysts show strong temperature-dependent selectivity (*e.g.*, CO *vs.* methanol *vs.* methane from CO_2_ + H_2_).^127^ In large industrial reactors, temperature gradients are inevitable, leading to different products forming in different reactor zones. This can reduce overall selectivity and complicate product separation.^128^

#### 2.8.5 Integration with renewable hydrogen

All CO_2_ hydrogenation processes require large amounts of H_2_. Currently, ∼95% of global H_2_ comes from fossil fuels (steam methane reforming), producing ∼10 tons of CO_2_ per ton of H_2_.^129^ Even the most efficient mechanochemical catalyst for CO_2_ reduction provides no net climate benefit if paired with (“grey” hydrogen).^130^ The transition to “green” hydrogen from water electrolysis powered by renewables is ongoing but currently accounts for <5% of production due to high costs ($4–6 per kg H_2_*vs.* $1–2 for grey H_2_).^131^ Coupling mechanochemically synthesized catalysts with renewable H_2_ requires co-location of electrolyzers, renewable energy sources (solar/wind farms), CO_2_ capture facilities, and conversion reactors.^132^ This integrated infrastructure is complex and capital-intensive, creating barriers to deployment especially in developing economies.^133^ Renewable energy is inherently intermittent (no sun at night, variable wind).^134^ CO_2_ conversion catalysts may need to operate in start-stop mode or at fluctuating loads, which can accelerate deactivation through thermal cycling and transient chemical environments. Mechanochemically synthesized catalysts optimized for steady-state conditions may perform poorly under such dynamic operation.^135^

## 3 Conclusion and future outlook

Mechanochemical synthesis has emerged as a game-changing approach for the development of advanced catalysts tailored for CO_2_ reduction. As this review has demonstrated, the technique offers significant advantages over conventional solution-based methods, including solvent-free operation, energy efficiency, scalability, and superior control over catalyst morphology and composition. A wide range of mechanochemically synthesized catalysts, spanning metal oxides, alloys, perovskites, single-atom catalysts (SACs), and hybrid systems, have shown remarkable performance across various CO_2_ conversion pathways, such as methanation, reverse water–gas shift, methanol synthesis, and even solid carbon fixation. Notably, exceptional CO_2_ reduction efficiencies have been reported, with the MgCuFe-BM catalyst achieving nearly complete CO_2_ conversion (∼98.5%) at 300 °C, underscoring the effectiveness of mechanochemical synthesis in producing highly active materials. Beyond enhancing catalytic activity and selectivity, mechanochemical methods also confer improved thermal stability, recyclability, and multifunctional integration for CO_2_ capture and conversion. These features are crucial for enabling long-term, sustainable applications in real-world environments. Moreover, the coupling of mechanochemical strategies with renewable hydrogen sources, continuous flow systems, and integrated reactor designs presents a compelling pathway for scalable green manufacturing. Despite these advances, challenges remain in terms of catalyst durability under fluctuating industrial conditions, mechanistic understanding at the atomic scale, and transition from laboratory-scale successes to industrial deployment. Future work should focus on the rational design of multicomponent catalysts, in-depth mechanistic studies using *operando* techniques, and the engineering of scalable mechanochemical reactors.

## Ethical statement

The researchers sought informed consent from all participants before recruitment for data collection.

## Consent for publication

The explicit consent for publication was also obtained from participants.

## Author contributions

Anam Shahzadi: writing – original draft. Muhammad Adnan Iqbal: conceptualization, resources, supervision. Adnan Majeed: writing review & editing, software. Iqra Yasmeen: data curation. Manahil Akmal: formal analysis. Sana Ejaz: software. Sabahat Fatima: software. Muhammad Nadeem Arshad: writing review & editing. Mohammad Asad: writing review & editing.

## Conflicts of interest

The authors declare that they have no competing interests.

## Abbreviations

a-ZrO_2_: Amorphous zirconia
BM: Ball milling
C1, C2, C_2_^+^: Carbon products containing one, two, or more than two carbon atoms
CBB: Cs_3_Bi_2_Br_9_
CO_2_RR: Carbon dioxide reduction reaction
CTAB: Cetyltrimethylammonium bromide
D–A: Donor acceptor
DAC: Direct air capture
DFM: Dual-functional material
EROEI: Energy return on energy invested
FTS: Fischer Tropsch synthesis
g-C_3_N_4_: Graphitic carbon nitride
HATNA-Cl_6_: Hexachloro-hexaazatrinaphthylene
HT: Hydrotalcite
ICCU: Integrated carbon capture and utilization
LUMO: Lowest unoccupied molecular orbital
MOF: Metal organic framework
MSI: Metal support interaction
RGO: Reduced graphene oxide
RT: Room temperature
RWGS: Reverse water gas shift reaction
SAC: Single-atom catalyst
SC: Sponge copper
SNAr: Nucleophilic aromatic substitution

## Data Availability

No primary research results, software, or code have been included and no new data were generated or analyzed as part of this review.
